# Supplementary material for: An open-source framework for large-scale, flexible evaluation of biomedical text mining systems
Source: J Biomed Discov Collab. 2008 Jan 29;3:1. doi: 10.1186/1747-5333-3-1 (PMC2276192; doi:10.1186/1747-5333-3-1)
Supplement: Additional file 1 — Evaluation platform source code. The additional file contains the software evaluation framework discussed in this paper. The most current release can be downloaded from SourceForge.net [1]. See the accompanying README file for instructions on installation and use. Also included in the distribution is a collection of UIMA wrappers for some commonly used BioNLP tools and annotated corpora. [file 1747-5333-3-1-S1.zip › bionlp_uima_repository-1.0.1/docs/api/src-html/edu/uchsc/ccp/uima/ae/nlp/sentenceDetector/OpenNLPSentenceDetector_AE.html]

```
001    /*
002     * Created on Aug 15, 2005
003     *
004     * I created this for Andy to test out SIAPI
005     */
006    package edu.uchsc.ccp.uima.ae.nlp.sentenceDetector;
007    
008    
009    import java.io.IOException;
010    import java.util.Iterator;
011    import java.util.List;
012    
013    import org.apache.uima.analysis_engine.ResultSpecification;
014    import org.apache.uima.analysis_engine.annotator.AnnotatorConfigurationException;
015    import org.apache.uima.analysis_engine.annotator.AnnotatorContext;
016    import org.apache.uima.analysis_engine.annotator.AnnotatorContextException;
017    import org.apache.uima.analysis_engine.annotator.AnnotatorInitializationException;
018    import org.apache.uima.analysis_engine.annotator.AnnotatorProcessException;
019    import org.apache.uima.analysis_engine.annotator.JTextAnnotator_ImplBase;
020    import org.apache.uima.jcas.JCas;
021    
022    import edu.uchsc.ccp.uima.annotation.CCPDocumentInformation;
023    import edu.uchsc.ccp.uima.util.UIMA_Util;
024    import edu.uchsc.ccp.util.external.opennlp.OpenNLPSentenceSegmenter;
025    import edu.uchsc.ccp.util.nlp.annotation.TextAnnotation;
026    
027    /**
028     * @author williamb
029     *
030     */
031    public class OpenNLPSentenceDetector_AE extends JTextAnnotator_ImplBase {
032            
033            
034            public static final String PARAM_MODEL_FILE = "OpenNlpModelFile"; 
035    
036        private OpenNLPSentenceSegmenter segmenter;
037        
038              public void initialize(AnnotatorContext arg0) throws AnnotatorInitializationException, AnnotatorConfigurationException {
039                 try {
040                  String modelFile = (String)arg0.getConfigParameterValue(PARAM_MODEL_FILE);
041              try {
042                segmenter = new OpenNLPSentenceSegmenter(modelFile);
043            } catch (IOException e) {
044                error("Error initializing OpenNLP sentence segmenter.");
045                e.printStackTrace();
046            }
047                    } catch (AnnotatorContextException e) {
048                            error("Error reading parameters from descriptor file.");
049                            e.printStackTrace();
050                    }
051                    super.initialize(arg0);
052                }
053            
054            
055            /* (non-Javadoc)
056             * @see com.ibm.uima.analysis_engine.annotator.JTextAnnotator#process(com.ibm.uima.jcas.impl.JCas, com.ibm.uima.analysis_engine.ResultSpecification)
057             */
058            public void process(JCas jcas, ResultSpecification arg1) throws AnnotatorProcessException {
059    
060            /* get document ID */
061            Iterator docInfoIter = jcas.getJFSIndexRepository().getAnnotationIndex(CCPDocumentInformation.type).iterator();
062            String documentID = "-1";
063            if (docInfoIter.hasNext()) {
064                CCPDocumentInformation srcDocInfo = (CCPDocumentInformation) docInfoIter.next();
065                documentID = srcDocInfo.getDocumentID();
066            }
067            
068                    /* get document text */
069                    String documentText = jcas.getDocumentText();
070            
071                    /* tokenized into sentences and words using oneline (part of the KeX system) */
072                    List<TextAnnotation> sentences = segmenter.segmentIntoSentences(documentText, documentID);
073                
074                /* add protein annotations to the CAS */
075                UIMA_Util uimaUtil = new UIMA_Util();
076                uimaUtil.putTextAnnotationsIntoJCas(jcas, sentences);
077                    
078            }
079        
080        private void error (String message) {
081            System.err.println("ERROR -- OpenNLPSentenceDetector_AE: " + message);
082        }
083        
084            
085            
086      
087    }
```
